# Ketonuria in an adult with Prader-Willi syndrome and diabetes mellitus: A case report

**DOI:** 10.1097/MD.0000000000037096

**Published:** 2024-01-26

**Authors:** Xiaoqing Xu, Dayang Wang, Huichai Pan, Jun Li, Bowu Li, Zhongchen He

**Affiliations:** a Department of Endocrinology, Beijing Hepingli Hospital, Beijing, China; b Institute of Cardiovascular diseases, Dongzhimen Hospital, Beijing University of Chinese Medicine, Beijing, China.

**Keywords:** case report, diabetes mellitus, ketonuria, Prader-Willi syndrome, sodium-glucose co-transporter 2 inhibitors

## Abstract

**Rationale:**

Prader-Willi syndrome (PWS) is a genetic disorder affecting multiple systems. Approximately one-quarter of PWS patients will develop diabetes. Given the uncontrolled hyperphagia and resultant severe obesity in these patients, their glycemic management poses a significant challenge.

**Case report:**

We present the clinical profile of a male patient diagnosed with both PWS and diabetes. Previous administration of the sodium-glucose co-transporter 2 (SGLT-2) inhibitor Canagliflozin resulted in improved glycemic control and weight management. But at the age of 25, the patient was hospitalized due to worsened glycemic control and the detection of ketonuria. After thorough examination and clinical observation, we discovered that the patient ketonuria was associated with enhanced lipid metabolism related to Canagliflozin. After excluding the risk of SGLT-2 inhibitor-induced euglycemic diabetic ketoacidosis, adjustments of the hypoglycemic regimen, building upon prior treatment, were recommended for the patient.

**Conclusion:**

It is important to note that among patients with both PWS and diabetes, the utilization of SGLT-2 inhibitors can lead to the emergence of ketonuria due to increased lipolysis. Therefore, any decision to discontinue SGLT-2 inhibitors should undergo thorough evaluation.

## 1. Introduction

Prader-Willi syndrome (PWS) is a genetic syndrome with an estimated prevalence of 1/10,000 to 1/30,000.^[1]^ Gene abnormalities leading to PWS includes paternally inherited deletion (65%–70%), maternal uniparental disomy (20%–30%), and imprinting defect (1%–3%) in a segment on chromosome 15 (15q11.2-q13).^[2]^ PWS is characterized by hypotonia and feeding difficulties in early infancy. However, patients in childhood and later stage show hyperphagia with food-seeking behavior and eventually develop morbid obesity. PWS will also present with developmental delay, short stature, cognition impairment, hypogonadism, and distinctive behavioral pattern (temper tantrums, stubbornness, manipulative and compulsive behaviors).

As a result of hyperphagia and obesity, the incidence of diabetes mellitus increased among PWS. The prevalence of diabetes in PWS was estimated as 7% to 24% in various studies.^[3–5]^ Due to limited evidence, there is no specialized therapeutic guideline targeting diabetes in PWS. However, metformin, alpha-glucosidase inhibitors, dipeptidyl peptidase-4 inhibitors (DPP-4 inhibitors), sodium-glucose co-transporter 2 inhibitors (SGLT-2 inhibitors), and glucagon-like peptide-1 receptor agonists (GLP-1RA) are available choices.^[6]^ We present a patient with PWS and diabetes who demonstrated ketonuria after using Canagliflozin. Reasons for ketonuria in this case are discussed, and we hope to provide insights in PWS treatment.

## 2. Case description

A 25-year-old Chinese male patient diagnosed with PWS was referred to our department. His height and weight at birth were 49 cm and 2700 g respectively. He had feeding difficulty owing to hypotonia during his first 2 years. However, at age 3, he presented with voracious appetite, obesity, and growth retardation. At age 10, he was biochemically diagnosed with diabetes, and PWS was later confirmed by genetic testing. He began to take metformin for diabetes, while growth hormone therapy lasted for 2 years and was then discontinued since his blood glucose was not well controlled. Despite attempts to manage his diabetes with other antidiabetic regimens, including insulin treatment, there was no significant improvement in blood glucose levels. At age 25, after 1 year of good compliance with Metformin (1500 mg/d), Sitagliptin (100 mg/d) and Canagliflozin (100 mg/d), he lost 20 kg; but his HbA1c was 9.22%, indicating unsatisfactory glycemic control. The patient had no family history of diabetes. Additionally, he was prescribed Acipimox (750 mg/d) for hyperlipidemia.

Upon admission, his BMI was measured at 23.8 kg/m², derived from a body weight of 62.5 kg and a height of 162 cm. His serum glucose was 10.4 mmol/L, accompanied by notable elevations in urine ketones (3+) and urine glucose (4+). Arterial blood gas analysis returned within normal parameters. Notably, biochemistry assessment indicated an elevated triglyceride concentration of 2.1 mmol/L (reference range < 1.7) and a heightened free fatty acid level of 1.1 mmol/L (reference range 0.1–0.6). While the patient reported excessive sweating, no other symptoms were reported.

**Figure 1.**
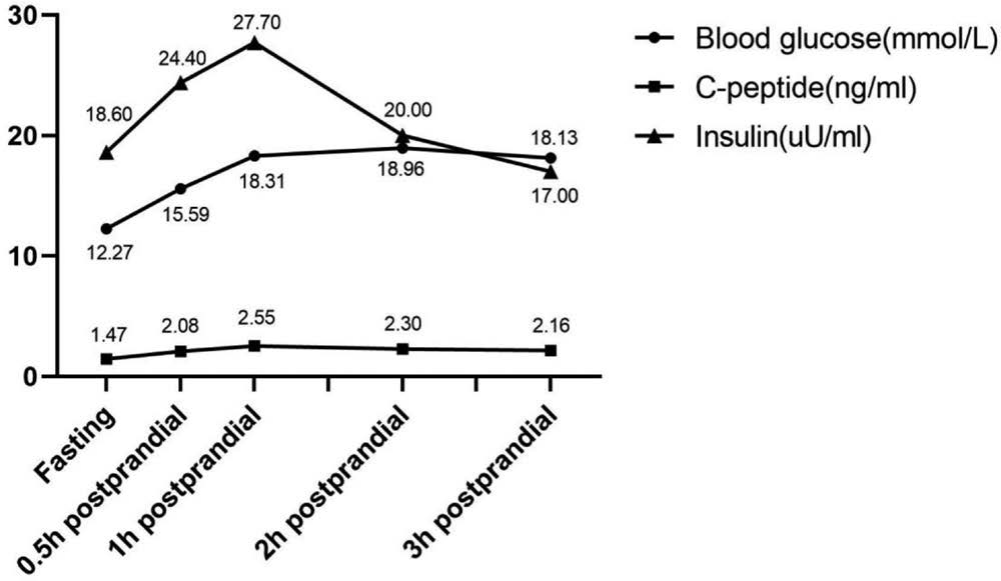
The oral glucose tolerance test (OGTT) result of this case. Patient blood glucose level was relatively high. The OGTT curve showed a marginally decreased level of C-peptide postprandially, suggesting a mild deficiency in insulin secretion after meals.

Further examinations yielded the following results: his thyroid function was in normal range, and islet-associated autoantibodies, islet cell antibodies, and glutamate decarboxylase antibodies were all negative. The oral glucose tolerance test outcome (refer to Fig. 1) revealed a mild impairment in insulin secretion. No sign of diabetic retinopathy, neuropathy, nephropathy, or peripheral vascular lesion was discovered. The Mini-Mental State Examination yielded a score of 26 out of 30, indicative of cognitive impairment.

While conclusive evidence of acidosis was not apparent, the diagnosis of diabetic ketosis was established based on the findings from his laboratory tests. Therefore, treatment of hydration and small-dose intravenous insulin infusion was administered. Then his urine ketone turned weakly positive.

To avert the progression of ketosis, the administration of his oral antidiabetic drugs was suspended, and transition was made to a basal-bolus insulin therapy regimen. This regimen encompassed the utilization of insulin glargine (Lantus) at a daily dosage of 10 units and insulin glulisine (Apidra) at 6 units per each meal. Concurrently, his dietary intake was regulated to 1500 kcal per day.

On the second day of hospitalization, his urine ketone levels increased to 2 + despite normal blood glucose levels. As a precautionary measure, he received intravenous hydration therapy once again. By the third day of hospitalization, urinary ketone bodies rose for the third time, reaching a level of 3+. Instead of administering fluid infusion, we advised the patient to enhance oral hydration.

Blood glucose readings during the hospital stay are outlined in Table 1. While initially adhering rigorously to the diet, the patient subsequently exhibited food-seeking behavior and hyperphagia. Notably, interventions involving dietary education and insulin dose adjustments failed to rectify the hyperglycemic state.

**Table 1:** Continuous glucose monitoring (mmol/L) during hospitalization.

D of hospitalization	FBG	Glu after breakfast	Glu before lunch	Glu after lunch	Glu before dinner	Glu after dinner	Glu before sleep
1st d			14.2	8.5	8.4	7.8	8.8
2nd d	6.1	12.8	9.9	9.9	8.4	11	9.8
3rd d	6.7	–	–	13.3	8.5	9.8	6.8
4th d	7.7	11.9	9.5	11.8	8.3	12.3	7.3
5th d	6.1	14.2	9.9	6.4	–	10.5	9.2
6th d	6.3	11.9	9.2	9.9	10.7	11.6	12.2
7th d	9.3	14.7	11.7	13.6	11.5	10.2	7.5
8th d	7.2	13	10.7	11.7	10.8	14	12.8
9th d	9.7	11.8	13.3	13.4	16.3	14.6	13
10th d	10.5	18.7	15	11.5	11.9	16.5	15.8
11th d	9.6	10.5	10.1	11	11.9	10.4	10.5

FBG, fasting blood-glucose, Glu, blood glucose.

Table 2 presents the urinalysis findings. His urine ketone turned negative on the 9th day post-admission. Following a comprehensive case discussion, it was concluded that ketonuria represented a physiological phenomenon rather than a pathological condition in this patient. Considering his poor blood glucose management and observed hyperphagia tendencies, we suggested him continue the regimen of Metformin and Canagliflozin, with the addition of GLP-1RA. However, following the initial administration of Dulaglutide (1.5 mg dosage, as the 0.75 mg dosage was unavailable), the patient reported abdominal distention and subsequently declined further doses.

**Table 2:** Urine ketone monitoring during hospitalization.

D of hospitalization	Urinalysis	Reexamination after treatment	Treatment
1st d	KET 3+	KET ±	Intravenous fluid therapy
2nd d	KET 2+	KET −	Intravenous fluid therapy
3rd d	KET 3+	KET −	Water intake
4th d	KET 1+		Undisposed
6th d	KET ± (in the afternoon)		Undisposed
7th d	KET 1+		Undisposed
9th d	KET −		Undisposed

KET, ketone.

Six months after discharge, his HbA1c deteriorated to 11.6%. Consequently, we recommended the initiation of Semaglutide therapy, starting titration with 0.25 mg/wk. However, due to concerns regarding potential gastrointestinal side effects, the patient abstained from escalating the dosage. Two months later, his HbA1c was 11.4% while fasting blood glucose levels lingered between 9 and 11 mmol/L. Then basal insulin treatment replaced Semaglutide, yielding a decline in fasting blood glucose to 8 to 9 mmol/L. His condition is still under follow-up.

## 3. Discussion

Diabetic ketoacidosis (DKA) is a life-threatening acute complication of diabetes. However, ketosis and acidosis do not always occur simultaneously owing to buffers in the blood. In individuals with diabetes, ketosis, often clinically identified through ketonuria, typically signifies the initial phase of DKA. This juncture necessitates prompt and appropriate intervention.^[7]^ Our major concern in this case was the potential risk of SGLT-2 inhibitor-induced DKA, although no symptom related to DKA was reported and arterial blood gas analysis was normal. DKA induced by SGLT-2 inhibitors commonly manifests as euglycemic DKA,^[8]^ entailing augmented fluid intake and a protracted acidosis recovery period.^[9]^ It is imperative to ascertain whether the detected ketonuria in this patient indicated an incipient stage of ketoacidosis. Additionally, the safety of sustaining the patient SGLT-2 inhibitor treatment warrants careful consideration.

It is well known that SGLT-2 inhibitors block glucose reabsorption in the proximal convoluted tubule, causing a relatively strong hypoglycemic effect. However, little was known that the hypoglycemic effect would subsequently promote gluconeogenesis that resembles starvation state, shifting glucose utilization to lipid utilization.^[10,11]^ Besides, the SGLT-2 inhibitor itself stimulates glucagon secretion,^[12,13]^ further enhancing lipolysis and ketogenesis. In vitro experiments verified that SGLT-2 inhibitors ameliorated obesity by increasing glucose uptake in skeletal muscle and lipolysis in adipose tissue; consequently, blood ketone levels in the SGLT-2 inhibitor group were raised.^[14,15]^ Under normal circumstances, the induction of ketosis by SGLT-2 inhibitors is precluded due to the regulatory feedback mechanisms in the human body.^[16,17]^ However, during conditions of fasting, surgery, acute infection, or insulin deprivation, the occurrence of DKA is markedly augmented.^[18]^

We noticed that this patient lost 20 kg after 1-year treatment with Canagliflozin; this magnitude of weight reduction far exceeded the average change in body weight typically associated with SGLT-2 inhibitor treatment.^[19]^ By promoting lipolysis, Canagliflozin helped to release the over-intake energy, generating excessive ketone. The elevated free fatty acid level also indicated enhanced lipid metabolism.^[20]^ Moreover, this patient had no DKA risk factors including inflammation, surgery, or islet failure. Based on evidence above, we concluded that it was safe for this patient to maintain SGLT-2 inhibitor treatment.

GLP-1RA are supposed to be ideal choices for PWS who have diabetes. The co-administration of GLP-1RA along with SGLT-2 inhibitors has been documented to effectively achieve targeted blood glucose control in multiple cases.^[21–23]^ Findings from randomized clinical trials have also indicated improved blood glucose levels attributed to GLP-1RA.^[24]^ In our presented case, poor adherence to prescribed medications emerged as the primary factor contributing to an inconspicuous response to GLP-1RA. This scenario posed a significant clinical challenge. Consequently, we deemed it necessary to initiate basal insulin therapy to effectively manage the patient condition.

## 4. Conclusion

In conclusion, for PWS with diabetes patients taking SGLT-2 inhibitors, ketonuria may be related to enhanced lipolysis, which should be carefully identified; and SGLT-2 inhibitors should not be easily withdrawn. PWS patients’ compliance will affect the use of GLP-1RA; in such situation, basal insulin treatment may benefit patients’ glucose control. Further studies are required to enact the therapeutic strategies for diabetes in PWS.

## Acknowledgments

The authors are very grateful to the patient for providing his case.

## Author contributions

**Data curation:** Huichai Pan, Jun Li, Bowu Li.

**Supervision:** Zhongchen He.

**Writing – original draft:** Xiaoqing Xu, Dayang Wang.
